# Diffusion MRI anomaly detection in glioma patients

**DOI:** 10.1038/s41598-023-47563-1

**Published:** 2023-11-21

**Authors:** Leon Weninger, Jarek Ecke, Kerstin Jütten, Hans Clusmann, Martin Wiesmann, Dorit Merhof, Chuh-Hyoun Na

**Affiliations:** 1 Department of Psychiatry, Psychotherapy and Psychosomatics, RWTH Aachen University, Aachen, Germany; 2 Present Address: Department of Electrical Engineering, RWTH Aachen University, Aachen, Germany; 3 Department of Neurosurgery, RWTH Aachen University, Aachen, Germany; 4 Center for Integrated Oncology Aachen Bonn Cologne Duesseldorf (CIO ABCD), Aachen, Germany; 5 Department of Neuroradiology, RWTH Aachen University, Aachen, Germany; 6 Faculty of Informatics and Computer Science, University of Regensburg, Regensburg, Germany; 7 Frauenhofer-Institut für Digitale Medizin, MEVIS, Bremen, Germany

**Keywords:** CNS cancer, Magnetic resonance imaging

## Abstract

Diffusion-MRI (dMRI) measures molecular diffusion, which allows to characterize microstructural properties of the human brain. Gliomas strongly alter these microstructural properties. Delineation of brain tumors currently mainly relies on conventional MRI-techniques, which are, however, known to underestimate tumor volumes in diffusely infiltrating glioma. We hypothesized that dMRI is well suited for tumor delineation, and developed two different deep-learning approaches. The first diffusion-anomaly detection architecture is a denoising autoencoder, the second consists of a reconstruction and a discrimination network. Each model was exclusively trained on non-annotated dMRI of healthy subjects, and then applied on glioma patients’ data. To validate these models, a state-of-the-art supervised tumor segmentation network was modified to generate groundtruth tumor volumes based on structural MRI. Compared to groundtruth segmentations, a dice score of 0.67 ± 0.2 was obtained. Further inspecting mismatches between diffusion-anomalous regions and groundtruth segmentations revealed, that these colocalized with lesions delineated only later on in structural MRI follow-up data, which were not visible at the initial time of recording. Anomaly-detection methods are suitable for tumor delineation in dMRI acquisitions, and may further enhance brain-imaging analysis by detection of occult tumor infiltration in glioma patients, which could improve prognostication of disease evolution and tumor treatment strategies.

## Introduction

Diffusion magnetic resonance imaging (dMRI) is a powerful technique that enables the measurement of molecular diffusion in biological tissues, providing valuable insights into tissue microstructure. In the context of brain imaging, dMRI allows for the characterization of nerve fibers and other microstructural properties of brain tissue. These measurements have proven to be useful in various neurological diseases, including brain tumors^1^, where significant alterations in tissue microstructure occur.

Despite the advancements in dMRI technology^2,3^, the current detection and delineation of brain tumors still heavily relies on conventional MRI techniques such as T1 and FLAIR acquisitions. These techniques have limitations in their ability to capture the intricate microstructural changes associated with brain tumors. Therefore, there is a need to explore the potential of dMRI in detecting microstructural anomalies and accurately delineating brain tumors.

In recent years, there has been significant progress in the field of supervised brain tumor segmentation using deep learning techniques. The Brain Tumor Segmentation (BraTS) challenge^4^, an international competition for automatic segmentation of brain tumor MRI scans, has played a pivotal role in driving advancements in this area^5^. State-of-the-art algorithms based on fully convolutional neural networks, such as the U-Net architecture, have achieved remarkable results, rivaling the performance of human annotators^6^.

The basis for the currently best performing supervised segmentation methods were laid out in 2015 with the fully convolutional neural networks^7^ and the U-Net^8^. While in 2016 only about 10% of the proposed algorithms for the BraTS challenge were deep learning-based, in 2020 it was over 95%^9^. For example, the current state-of-the-art, i.e., the winning algorithm of 2021, is an optimized U-Net with an open-source network architecture design^10^. It is very similiar to the nnU-Net developed by Isensee et al.^11^, which won the previous year’s challenge. Compared to the original U-Net, modifications focus on post-processing, region-based training, and data augmentation.

Detection and segmentation of brain tumors directly on dMRI data heretofore mostly relied on diffusion-derived scalar maps or combination of dMRI measurements with other MRI acquisitions. In particular, it was shown that incorporating diffusion tensor image (DTI) data into brain tumor segmentation improves the segmentation accuracy^12^. It is also possible to delineate tumor volumes of interest using only DTI data and to use the resulting volumes for tumor classification^13^. Further, deep learning tumor segmentation on apparent diffusion coefficient (ADC) maps is improved when distortion-correction is used^14^. ADC maps can also be used in combination with other MRI images and a deep learning approach to characterize brain tumors^15^.

In contrast to supervised brain tumor segmentation, anomaly detection methods model the healthy distribution of brain shape and tissue properties and identify out-of-distribution samples^16^. As MRI scans are currently unrivaled in detailing soft tissue, MRI is mostly the modality of choice for brain tumor anomaly detection^17^. Anomaly detection methods for the human brain are often based on Autoencoders^18^ or Generative Adversarial Networks (GANs)^19^.

However, off-the-shelf anomaly detection methods are not optimally suited for medical data. Special attention should be paid to two aspects: First, if a medical measurement is anomalous, it is often not only dependent on the instantaneous value, but often dependent on external factors such as the environment and history. For example, an increased heartbeat may indicate a disease, but may be completely normal during exercise. Similar effects are observed in medical image data, where certain value ranges may be normal or abnormal depending on the age and medical history of the patient. Secondly, the sensitivity, i.e. the true positive rate, is often of greater importance in medicine than in industrial applications. Thus, medical anomaly detection methods have unique properties not found in other application settings^20^.

A variety of brain anomaly detection methods have been proposed in recent years. Baur et al.^21^ compared a variety of autoencoder architectures to model structural brain MRI scans of healthy subjects. Using the trained models, a residual map was generated and multiple sclerosis lesions were delineated. It was concluded that the AnoVAEGAN architecture is best suited for the task. Zimmerer et al.^22^ augmented a similar variational autoencoding architecture with a context-encoding branch, and used the residual map to segment brain tumors. They also performed a systematic hyperparameter study to find the optimum training and application setting. In effect, they showed that the reconstruction error is improved by including a Kullback-Leibler-loss.

Two of the latest approaches in the field of anomaly detection on brain MRI data deal with highly advanced VAEs. Marimont et al.^23^ combined density and restoration-based approaches using Vector-Quantized Variational Auto-Encoders (VQ-VAE). They were able to achieve better results compared to a conventional VAE on the MOOD dataset, which consists of brain MR and abdominal CT images. Furthermore, by combining a VAE with a Transformer, Pinaya et al.^24^ improved pixel-wise detection of brain MRI datasets with small vessel disease.

Algorithms that rely on groundtruth annotations outperform unsupervised methods on current benchmarks^25^. Nevertheless, unsupervised methods that are able to delineate tumors without being trained on prior annotations are better suited to deal with changing acquisition parameters and offer a protection against unusual effects not seen in the training database. Further, as dMRI acquisitions offer unmatched insights into brain tissue microstructure, we hypothesize that this acquisition method can be used to detect tumor-induced brain changes earlier compared to conventional methods.

Therefore, in this study, we leverage deep learning-based anomaly detection methods to detect and analyze brain tumors in dMRI data. We compare the performance of different deep learning models against a groundtruth segmentation obtained from structural MRI scans. Trained neurooncologists also inspect the results, focusing on areas where the groundtruth segmentation and the anomaly detection method deviate. Furthermore, we investigate the correlation between anomaly scores and follow-up data from the same patients to explore the potential of anomaly scores as biomarkers for early glioma infiltration.

By harnessing the unique capabilities of dMRI and deep learning-based anomaly detection, our study aims to advance the field of brain tumor detection and contribute to improved diagnosis and treatment planning for brain tumor patients. To promote transparency and enable further research, we have made the code implementation of our anomaly detection model publicly available on GitHub at https://github.com/JarekE/Anomaly-detection-in-diffusion-MRI-for-brain-tumor-patients.

## Materials and methods

### Data

Two datasets were used for this work:

**Brain tumor dMRI dataset** The first dataset consists of dMRI scans of cerebral gliomas, acquired at the University Hospital Aachen (UKA). 32 patients and a control group of 28 age- and sex-matched healthy subjects were enrolled in the study. All subjects gave written informed consent to participate in the study. The study was approved by the local ethics committee of the Medical Faculty of the University of the RWTH Aachen (EK294-15) and conducted in accordance with the standards of Good Clinical Practice and the Declaration of Helsinki. Each dataset consists of a T1, FLAIR and a dMRI acquisition. The dMRI data are single-shell acquisitions with b-value = 1000 s $\hbox {mm}^{-2}$, one b-value = 0 s $\hbox {mm}^{-2}$, TE = 81 ms, TR = 6300 ms, anterior-posterior phase encoded, 64 gradient directions, an isotropic voxel size of 2.4 $\hbox {mm}^{3}$ and $90 \times 90 \times 54$ voxels. The dMRI acquisitions were corrected for susceptibility-induced correction with the FSL TOPUP toolbox^26^ as described in^27^, and for eddy currents and motion artifacts with FSL EDDY^28^. The reverse-phase encoded image was wrongly acquired or corrupted in five cases. In these cases, only FSL EDDY was applied. Then, the brain was extracted using FSL BET^29^. The T1 and FLAIR images were used to obtain a groundtruth tumor segmentation (see section “Groundtruth segmentation” for details). Finally, the anatomical T1 weighted images were registered to the pre-processed dMRI b0 images using symmetric diffeomorphic image registration as implemented in ANTs^30^, and the tissue segmentations and parcellations (including segmentation of the ventricles which were excluded from the analysis) were transformed into the diffusion space. The proposed anomaly detection algorithms were trained on the healthy control group, anomaly detection was performed on the patient images.

Follow-up data of 28 of the 32 patients was available, consisting of a FLAIR-weighted MRI acquisition. The median time interval between preoperative scan and follow-up scan is 17.1 months ($\pm \, 7$).

**BraTS dataset **Images from 1251 brain tumor patients-data including T1, T1 post-contrast, T2 and FLAIR acquisitions as well as a tumor segmentations-were extracted from the BraTS initiative. The tumor segmentation comprises the classes necrosis, enhancing tumor, and edema. It was determined by previous top-ranked BraTS algorithms and manually refined by neuroradiology experts^4^. The MRI scans were acquired with different protocols and various scanners from multiple institutions. The images were resampled to a uniform isotropic resolution of 1 $\hbox {mm}^{3}$, and preprocessed as well as skull-stripped with the Cancer Imaging Phenomics Toolkit (CaPTk)^31^.

### Groundtruth segmentation

To obtain a groundtruth tumor segmentation on the *brain tumor dMRI dataset*, a customized supervised deep learning network was trained on the *BraTS dataset*. The supervised segmentation framework was based on the optimized U-Net for brain tumor segmentation^10^. To repurpose the network for this work, minor changes had to be made. Specifically, the number of input images was reduced and the network was trained on T1 and FLAIR images only. For preprocessing, the BraTS data was freed from redundant background voxels, normalized and a one-hot encoding mask of the foreground voxels was created to distinguish values close to zero from the background. The mask was stacked with the input data.

The T1 and FLAIR images of the brain tumor dMRI dataset were identically preprocessed and spatially adjusted. After the optimized U-Net inferred the brain tumor segmentations, they were transformed into the diffusion space by registration of the T1 to DTI-derived fractional anisotropy maps using the ANTs toolbox^30^. Nearest-Neighbor interpolation was applied to transform the segmentations. Due to the lower resolutions of the second images, spurious intermediate values would otherwise occur. The tumor segmentations in the dMRI space were finally checked for plausibility by trained experts.

As expected, our network with only two MRI sequences as input was inferior to the original network. On a separate BraTS validation dataset, we reach dice scores of 0.89. The original network with all four input channels reaches dice scores of 0.92.

### Anomaly models

Several different anomaly detection frameworks exist. We investigated the two most promising deep learning approaches. While both are based on state-of-the-art techniques, they differ in their fundamental principles. These fundamental anomaly detection principles were previously published for MRI tumor detection as well as for visual anomaly detection. Here, we adapt these fundamental principles to dMRI data and modify them for our setting. All models were trained unsupervised only on dMRI data of the healthy subjects found in the brain tumor dMRI dataset.

#### Denoising autoencoder

**Figure 1:**
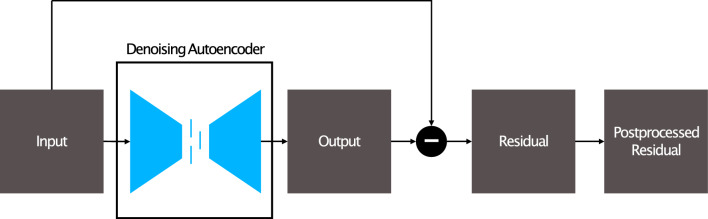
Setup of the denoising autoencoder. The network is trained using the control group with the aim to learn a reconstruction of the healthy brain. Finally, the residual is formed and post-processed.

The first principle is to use a denoising autoencoder (DAE) for anomaly detections. The DAE is an architecture that is designed to reconstruct corrupted data. While they are traditionally used for noise reduction, the denoising effect can be used for anomaly detection. As the network is only able to reconstruct known patterns, anomalies in the input data result in a faulty reconstruction. In our case, brain tumors should be transformed into healthy tissue, and this anomaly can be detected based on the difference between input and output. It is important to note, that this holds only if the model has been trained exclusively on healthy images. Figure 1 pictures this general framework. Subtracting the input from the output leads to a 3D anomaly score map.

Here, a convolutional variational autoencoder architecture with a linear latent space was chosen. We varied the latent space dimension (LSD) to evaluate the optimal amount of information for reconstruction. The network should be able to reproduce the input data in different variations of detail.

The architectural details were as follows: Each convolutional layer was normalized and activated by a LeakyReLU function with a negative slope of 0.01. The loss of the model was composed of the *mean squared error (MSE)* and the *Kullback-Leibler (KL) divergence* as:

1 $$\begin{aligned} Loss = MSE + \omega \cdot \left[ - \frac{1}{2} \cdot \frac{1}{n} \cdot \sum _{j}^{n} (1 + \log {\Sigma (x_j)} - \mu (x_j)^2 - \Sigma (x_j) )\right] \end{aligned}$$

with $x_j$ elements of the latent space vector with size *n*. The KL divergence was only slightly weighted with $\omega = 1.22 \cdot 10^{-5}$.

Due to the high amount of possible combinations and limited computing power, we could not test every possible set of hyperparameters. Thus, for the following set of experiments, we needed to decide on a set of reasonable hyperparameters without an extensive hyperparamter search. The mentioned loss weight was chosen to put the focus on the reconstruction.

Adam, together with a learning rate scheduler, was used as an optimizer. The learning rate per epoch was decayed by $\gamma = 0.95$ and the model was trained for 500 epochs. Furthermore, a batch size of 4 was chosen. With the most computationally intensive parameter configuration, the network could be trained with 20 GB VRAM, most configurations with less.

To evaluate this approach, the residual *R* of the output and input data were formed. As the anomaly detection was carried out on the raw diffusion images, the output comprised 64 feature maps. We calculated the residual per channel for each voxel, summed the residuals up and took the absolute value after summation. Taking the absolute value after the summation reduced noise in the individual channels. The residual map was composed by all calculated residual voxels. Finally, the classification into binary anomaly maps was obtained through a threshold. Details on the setting of this threshold can be found in section “Evaluation”.

With DAEs for anomaly detection, noise may lead to an incorrect reconstruction and a misclassification of voxels. We reduced this misclassification and ensured a spatial consistency through morphological opening of the binary images. A cubic kernel with side length 3 was used as structure element.

To optimize the reconstruction quality of the network, two parameters were optimized. First, the **output activation functions**. Here, two different options were compared. The sigmoid function maps to the value set of the input data (0, 1) and is often used for binary classifications. As an alternative, a linear output function was tested. As a second parameter, the amount of information for reconstruction was modified in each case. Different dimensions of the linear **latent space** vector *x* were tested for the DAE. The sizes 16, 32, 64, 128, 256 and 512 were considered. In general, it can be assumed that a smaller latent space makes the reconstruction more difficult. A table detailing the architecture can be found in the supplementary materials S1.

#### Discriminatively trained reconstruction anomaly embedding

**Figure 2:**
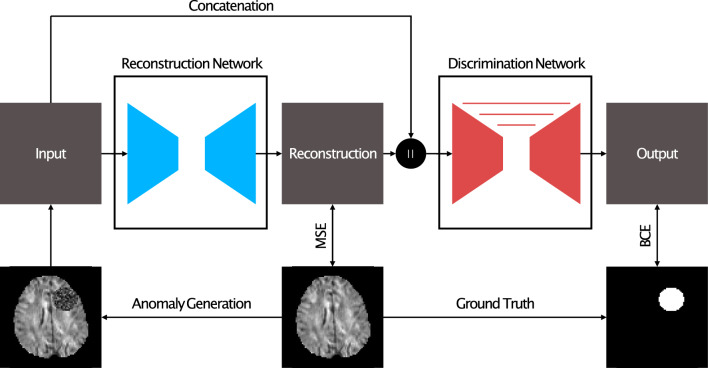
Images of the control group are enhanced by just-out-of-distribution appearances. The reconstruction network is trained to denoise the input images using the mean squared error, while the discrimination network utilizes the concatenated images of input and reconstruction to create an anomaly map. The artificial groundtruth and a weighted Binary Cross Entropy loss is applied to train the discrimination network.

The second approach is based on the discriminatively trained reconstruction anomaly embedding model (RecDiscNet)^32^. By using a two-step approach, with a DAE as a first step and a discrimination network as a second step, as well as synthetically adding just-out-of-distribution appearances to the input data, we hypothesized that a stronger learning effect can be achieved. It should be noted that there was still no need for labeled data or simulations that reflect the real anomaly appearance in the target domain. Figure 2 visualizes this approach.

As a reconstruction network was trained to denoise the artificially altered input images while the discrimination network tried to detect the anomaly using the concatenated input and reconstruction data, further preprocessing of the training data was necessary. Artificial noise needed to be added to the input data and a groundtruth segmentation map needed to be generated. A random number of anomaly patches was applied to each input image. 60% of the images were inpainted with one patch and 20% each with none or two. All patches were ellipsoids that varied in size, orientation, and axis projections. They were placed exclusively in the brain but overlapping of different tissue types or background was possible. Spatial information was applied equally to all channels. For data augmentation, each dataset was expanded to four input images by including different patches.

Our proposed dMRI just-out-of-distribution appearances were based on heuristics. Different methods of generating and incorporating these appearances were evaluated. The proposed appearances were based on random distributions and are scanner- and tumor-independent.

**Figure 3:**
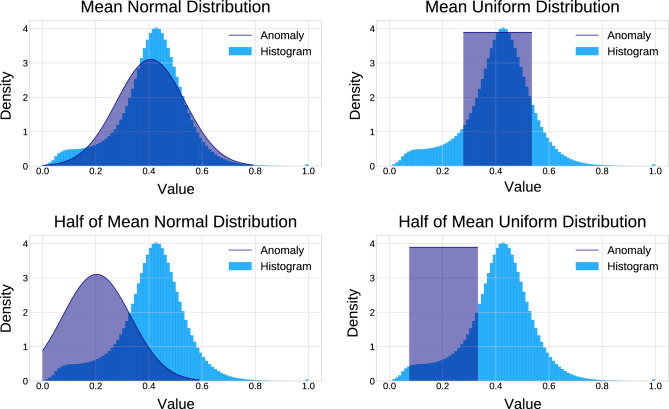
Normal and uniform distribution, defined by the mean and standard deviation of observed MRI signal diffusion attenuation in the brain. The noise spectra are also shown with the mean value halved.

Different random distribution characteristics were evaluated. The noise was either sampled from a normal distribution with the mean and standard deviation set to the mean and standard deviation of brain tissue or to half the mean and the standard deviation of the brain, or sampled from a uniform distribution where the mean value was set to the mean or half the mean value of the brain, with the range chosen as the standard deviation of brain tissue. A visual representation of possible noise distributions is given in Fig. 3.

In the noise block-consisting of four dimensions-each voxel was either (1) assigned a randomly sampled value, or (2) a random value was fixed per diffusion-attenuated image, leading a spatially consistent but channel-specific block, or (3) a random value for every voxel location was fixed, resulting in a random value that is the same across all diffusion directions. These three approaches can be understood as random (1), directionally-dependent (2) and isotropic out-of-distribution diffusion (3).

The loss of the model was composed of two parts. The reconstruction was evaluated using the MSE, whereas the discrimination was interpreted using the Binary Cross Entropy (BCE) loss:

2 $$\begin{aligned} Loss = MSE + \omega \cdot \left[ - \frac{1}{n} \cdot \sum _{i}^{n} (p_1 \hat{y}_i \cdot \log {\sigma (y_i)} + (1 - \hat{y}_i) \cdot \log {(1 - \sigma (y_i))} )\right] \end{aligned}$$

The implementation presented here includes the sigmoid activation function $\sigma (y)$. The second part measures the *BCE* between the target $\hat{\textbf{y}}$ and the input $\textbf{y}$ probabilities, whereby n is the number of data points. Furthermore, two weights were introduced. On the one hand, $\omega$ amplifies the impact of *BCE* on the total loss. $p_1$, on the other hand, weights the positive class. This compensated for class imbalances in the anomaly map. The optimizer, batch size and number of epochs was chosen accordingly to the DAE. The model presented here required a GPU with at least 24 GB VRAM.

### Evaluation methods

The anomaly maps of the models were evaluated by utilizing the residual map for the DAE and the output for the RecDiscNet. To avoid artificially improving the results, the evaluation was confined to areas within the brain mask. For this purpose, only voxels belonging to the white or gray matter of the brain were considered.

The results were quantified by two classical metrics for tumor detection: The Dice coefficient—the most commonly used metric in most segmentation research, especially in the BraTS challenge—is used for binary map evaluation. The receiver operating characteristic (ROC) curve and the corresponding area under the ROC curve (AUC) are used to evaluate anomaly maps, as the AUC value provides a performance metric across all possible thresholds.

For evaluation, the anomaly map was further post-processed to a binary map. We used different approaches to select the classification threshold. The simplest approach is a pre-fixed value. Thus, the treshold was set to 0.5 for the RecDiscNet and to 0.3 for the DAE. The first value results from halving the discrimination map output range and the latter pursues the idea to detect even small deviations in the residual of the DAE. Furthermore, the Otsu method for threshold selection was evaluated. Lastly, we determined a threshold by pooling the output image *I* with a $16\times 16\times 16$ local average kernel $f_{local}$ and selecting the resulting global maximum value:

3 $$\begin{aligned} T = \max (I * f_{local}) \end{aligned}$$

Moreover, supervised methods for threshold selection were evaluated. In this context, supervised implies that information about the anomaly map and the corresponding groundtruth were available. Thus, it must be noted that these results should only be utilized for comparing the outputs of different anomaly model architectures. The first supervised threshold was based on the ROC Curve. For this, the threshold *T*, which maximizes the subtraction of True Positive Rate and False Positive Rate, was selected. This is also called maximum value of Youden’s index^33^. Another approach used here is to choose the threshold that optimizes the binary Dice coefficient.

All results were evaluated with 7-fold cross validation. Therefore, the 28 healthy subjects in the training datasets were divided into 7 random subsets, which is a trade-off between the number of subsets and generality per subset. Each subset was used once as validation set. All network parameter and callbacks were chosen by the validation loss only.

## Results

### Evaluation of the anomaly map

We first evaluated the performance of the anomaly detection models by calculating the AUC for different configurations of DAE. Table 1 presents the AUC values for various parameter combinations, including the LSD and the output activation function (linear or sigmoid). We observed that models using the sigmoid activation function achieved better results compared to those with the linear function. While the model with the largest latent space dimensionality achieved the highest AUC of 0.820 ± 0.11, even the smallest model performed relatively well with an AUC of 0.804 ± 0.12.

**Table 1:** Area under the receiver operating characteristic curve (AUC) of the DAE models.

*m*	LSD	Activation	AUC
1	256	Linear	0.739 ${}\pm {}$ 0.12
2	64	Linear	0.765 ${}\pm {}$ 0.11
3	128	Linear	0.765 ${}\pm {}$ 0.11
4	32	Linear	0.771 ${}\pm {}$ 0.11
5	16	Linear	0.779 ${}\pm {}$ 0.11
6	512	Linear	0.793 ${}\pm {}$ 0.11
7	128	Sigmoid	0.803 ${}\pm {}$ 0.12
8	64	Sigmoid	0.804 ${}\pm {}$ 0.12
9	16	Sigmoid	0.804 ${}\pm {}$ 0.12
10	32	Sigmoid	0.808 ${}\pm {}$ 0.11
11	256	Sigmoid	0.818 ${}\pm {}$ 0.11
**12**	**512**	**Sigmoid**	**0.820** ${}\pm {}$ **0.11**

While the activation function of the output layer has a major impact on the results, the latent space dimension (LSD) is secondary.

Bold values indicate the maximum values.

For the RecDiscNet, we assessed the AUC values for different combinations of distribution types, appearance types, and mean values of disturbances. It should be noted that calculating a residual was not necessary, the output of the RecDiscNet was directly used as anomaly map. Table 2 presents the results, showing that models trained with a mix of all available parameters achieved the highest AUC of 0.772 ± 0.17. The choice of the just-out-of-distribution disturbance had a strong effect on the results. In particular, models trained with a $\mu _{Half}$ disturbance outperformed those trained with $\mu _{Full}$ disturbance. Overall, the RecDiscNet demonstrated good performance in anomaly detection.

**Table 2:** The area under the receiver operating characteristic curve (AUC) of the RecDiscNet for different just-out-of-distribution appearances parameter combinations.

*d*	Distribution	Type	$\mu$	AUC
1	Normal	Directional	Full	0.550 ${}\pm {}$ 0.18
2	Uniform	Directional	Full	0.565 ${}\pm {}$ 0.19
3	Uniform	Random	Full	0.568 ${}\pm {}$ 0.18
4	Normal	Random	Full	0.594 ${}\pm {}$ 0.20
5	Mix	Mix	Full	0.660 ${}\pm {}$ 0.21
6	Uniform	Random	Half	0.673 ${}\pm {}$ 0.17
7	Uniform	Directional	Half	0.680 ${}\pm {}$ 0.16
8	Normal	Random	Half	0.693 ${}\pm {}$ 0.15
9	Uniform	Isotropic	Full	0.700 ${}\pm {}$ 0.15
10	Uniform	Isotropic	Half	0.761 ${}\pm {}$ 0.16
11	Normal	Directional	Half	0.763 ${}\pm {}$ 0.14
**12**	**Mix**	**Mix**	**Half**	**0.772** ${}\pm {}$ **0.17**

A clear difference can be observed in the mean $\mu$, with halving showing clear improvements. The best result is achieved with a *Mix* of all available parameters. In general, the AUC is lower compared to top performing denoising autoencoder models.

Bold values indicate the maximum values.

### Distribution of anomaly scores

**Figure 4:**
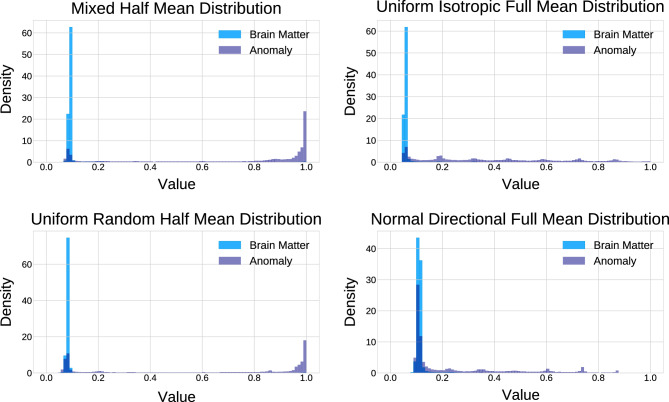
The distribution of the best (top) and worst (bottom) performing model for $\mu _{Half}$ (left) and $\mu _{Full}$ (right) is shown. The models with $\mu _{Half}$ create a clear separation of brain matter and anomalies. The worst model behaves similarly to a random classifier, which fits to an area under the receiver operating characteristic curve of 0.55.

To gain further insights into the discriminatory performance, we analyzed the density distributions of anomaly scores generated by the best and worst performing models in Fig. 4. We observed that brain matter was mostly scored close to zero by all models, while the scoring of anomalies varied. The best performing model (top left, d = 12) exhibited a strong separation between anomalies and healthy brain tissue, indicating its ability to effectively detect anomalous regions. In contrast, the worst performing model (bottom right, d = 1) showed no clear separation between anomalies and healthy tissue.

### Binary segmentation

We compared the performance of the DAE and the RecDiscNet in binary segmentation of brain tumors and presents the Dice scores obtained using different thresholding methods. All tables presented here show the best performing model configurations for DAE and the RecDiscNet. *Dice Postprocessed* means that the images have been processed with morphological opening between thresholding and calculating the Dice score.

Table 3 shows the Dice score for the different models that are achieved with the pre-defined threshold method. The RecDiscNet achieved the best result with a Dice score of 0.603 ${}\pm {}$ 0.24. Post-processing clearly improves the results of the DAE, achieving a Dice score of 0.431 ${}\pm {}$ 0.19.

As a pre-defined threshold may be suboptimal, two data-driven thresholding methods were evaluated, the Otsu threshold and the maximum value of the mean pooled output treshold selection (see section “Materials and methods” for details).

**Table 3:** Dice scores obtained using the pre-defined threshold.

Network	Index	Threshold	Dice	Dice postprocessed
DAE	4	0.3	0.168 ${}\pm {}$ 0.11	**0.431** ${}\pm {}$ **0.19**
RecDiscNet	10	0.5	**0.603** ${}\pm {}$ **0.24**	0.599 ${}\pm {}$ 0.25

Bold values indicate the maximum values.

The Otsu threshold strongly improved the results of the DAE with a Dice score of 0.501 ${}\pm {}$ 0.20 (Table 4). It should be noted that the calculated thresholds for DAE were lower than the previous assumption of 0.3. However, the RecDiscNet is not improved by Otsu, as the determined threshold is very close to the pre-defined fixed threshold.

**Table 4:** The Otsu threshold improves the results, especially for the post-processed DAE.

Network	Index	Threshold	Dice	Dice postprocessed
DAE	4	0.18	0.195 ${}\pm {}$ 0.13	**0.501** ${}\pm {}$ **0.20**
RecDiscNet	10	0.50	**0.603** ${}\pm {}$ **0.24**	0.598 ${}\pm {}$ 0.25

For d = 10 almost the vanilla value is determined.

Bold values indicate the maximum values.

Determining the treshold with the maximum value of the mean pooled output, all results were improved (Table 5). The post-processed scores were superior for both models, with strongly increasing Dice score for the DAE.

**Table 5:** The maximum of the mean pooling image is based entirely on the output data and provides a good basis for one-class segmentation of anomalies.

Network	Index	Threshold	Dice	Dice postprocessed
DAE	11	0.36	0.230 ${}\pm {}$ 0.18	**0.483** ${}\pm {}$ **0.24**
RecDiscNet	10	0.31	0.616 ${}\pm {}$ 0.24	**0.624** ${}\pm {}$ **0.24**

The post-processed results are superior here, with the RecDiscNet scoring the highest.

Bold values indicate the maximum values.

Finally, we present the optimal threshold, i.e., the threshold optimized knowing the groundtruth data. For this purpose, the threshold that results in the best Dice coefficient without post-processing was used. Table 6 shows the results for the test data. As expected, the Dice score increased in comparison to the data-driven thresholds for both models. The RecDiscNet still performed best with Dice = 0.660 ${}\pm {}$ 0.23.

**Table 6:** Optimal results: the dice score was chosen such that the unprocessed dice score is optimized.

Network	Index	Threshold	Dice	Dice postprocessed
DAE	12	0.49	0.375 ${}\pm {}$ 0.18	**0.519** ${}\pm {}$ **0.18**
RecDiscNet	12	0.23	0.657 ${}\pm {}$ 0.23	**0.660** ${}\pm {}$ **0.24**

Bold values indicate the maximum values.

### Qualitative comparison

This section shows the qualitative output of the presented models. Figures 6, 7 and 8 each demonstrate the ability to detect a *Large*, *Medium* and *Small* tumor of different models. In these figures, the column *Data* shows the mean diffusivity image and groundtruth segmentation. The colorbar refers to the anomaly *Map* only, while the unprocessed *Binary* and *Postprocessed* images represent the binary segmentations using the respective threshold.

**Figure 5:**
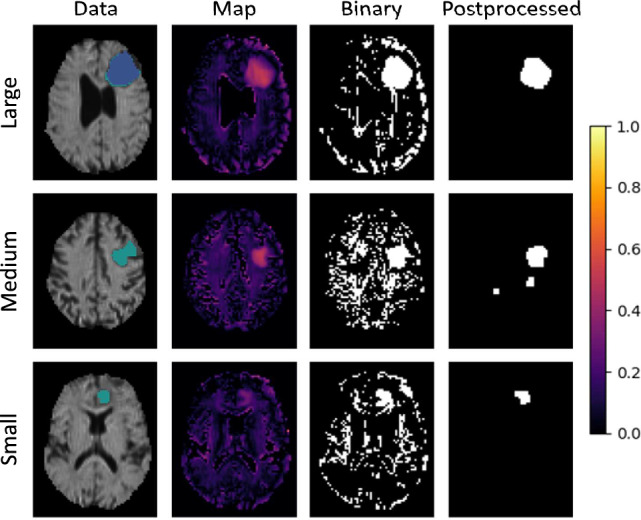
Qualitative results of the DAE model m = 4 with the Otsu threshold.

The results of the best performing DAE model for the unsupervised threshold can be seen in Fig. 5. The quantitative Dice scores can be recognized here. While the model was able to generate solid segmentations after post-processing, the unprocessed images were very noisy. The single misclassified voxels at the transitions to the CSF were well visible and were eliminated by filtering.

**Figure 6:**
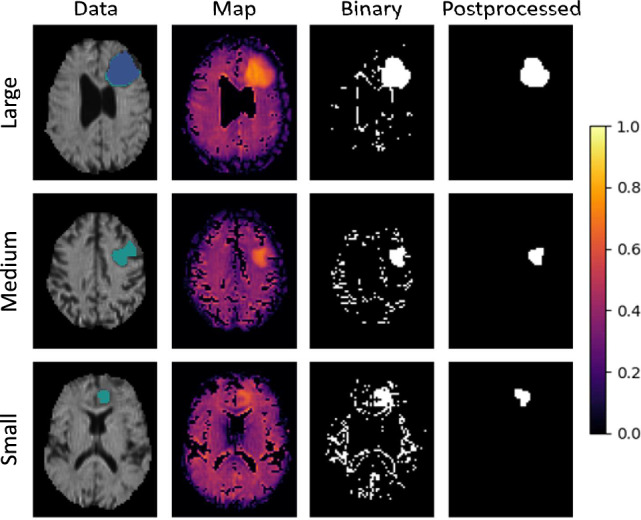
Qualitative results of the DAE with a supervised threshold.

The best Dice score for DAE were achieved by model m = 11. Figure 6 therefore shows the qualitative results with the optimized threshold. The quality of the processed results in particular was greatly improved here. There was less background noise, but smaller tumors were still difficult to detect.

**Figure 7:**
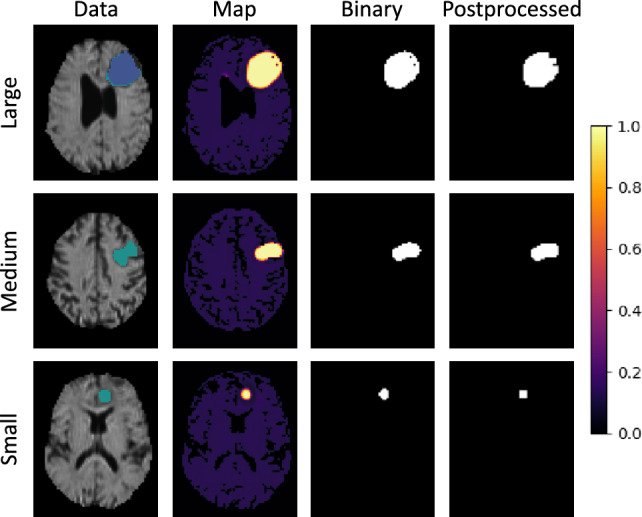
Qualitative results of the RecDiscNet d = 10. Using an unsupervised threshold, excellent results can be achieved even without post-processing.

Corresponding to the Dice scores, the qualitative results of the RecDiscNet consistently exhibited superior performance. Figure 7 shows d = 10 with the maximum mean pooling threshold for both maps. Here, the post-processed image differed only minimally. It can be stated that the RecDiscNet achieved excellent qualitative results without post-processing and without a supervised threshold value. The post-processing may even slightly degrade the excellent quality of the results in some cases, as can be seen for the RecDiscNet in combination with the “large” tumor patient.

**Figure 8:**
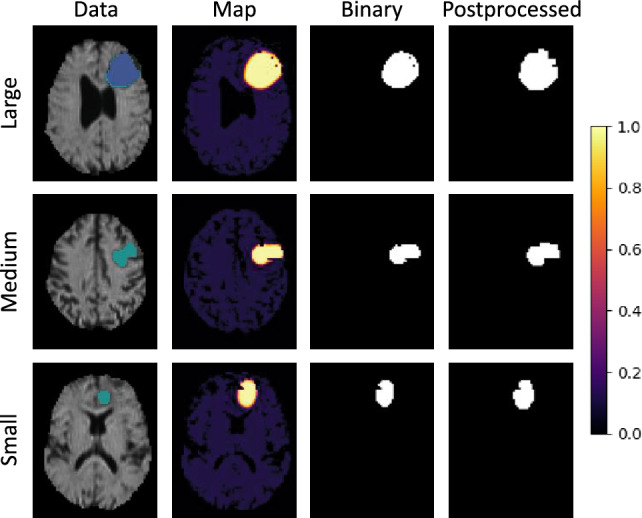
RecDiscNet d = 12 with a supervised threshold. The qualitative results are comparable to those with the maximum mean pooling threshold.

Finally, Fig. 8 shows the qualitative maps of the quantitatively optimized network, the RecDiscNet d = 12 with a supervised threshold. Here, too, excellent results were achieved, comparable to those shown before.

### Visual comparison of diffusion anomalies with follow-up scans

Finally, particular attention was given to areas marked by the anomaly models that were not present in the groundtruth segmentation maps. While these areas could be due to faulty segmentation, it is also possible that the anomaly detection algorithm highlights early changes of the brain structure not yet delineated in conventional MRI. To test this hypothesis, mismatching areas of the groundtruth tumor segmentations with anomaly-score maps were further evaluated by comparison to preoperative as well as post-operative structural MRI follow-up scans.

While the RecDiscNet showed superior discriminative ability between tumor and healthy tissue with superior Dice scores providing a clear distinction into normal and abnormal tissue (see Figs. 5, 6, 7 and 8), the DAE models achieved better AUROC scores (see Tables 1 and  2) as their output introduces some uncertainty of the anomaly scoring by capturing as well areas of continuous transition. The best DAE model was thus used for further visual inspection.

**Figure 9:**
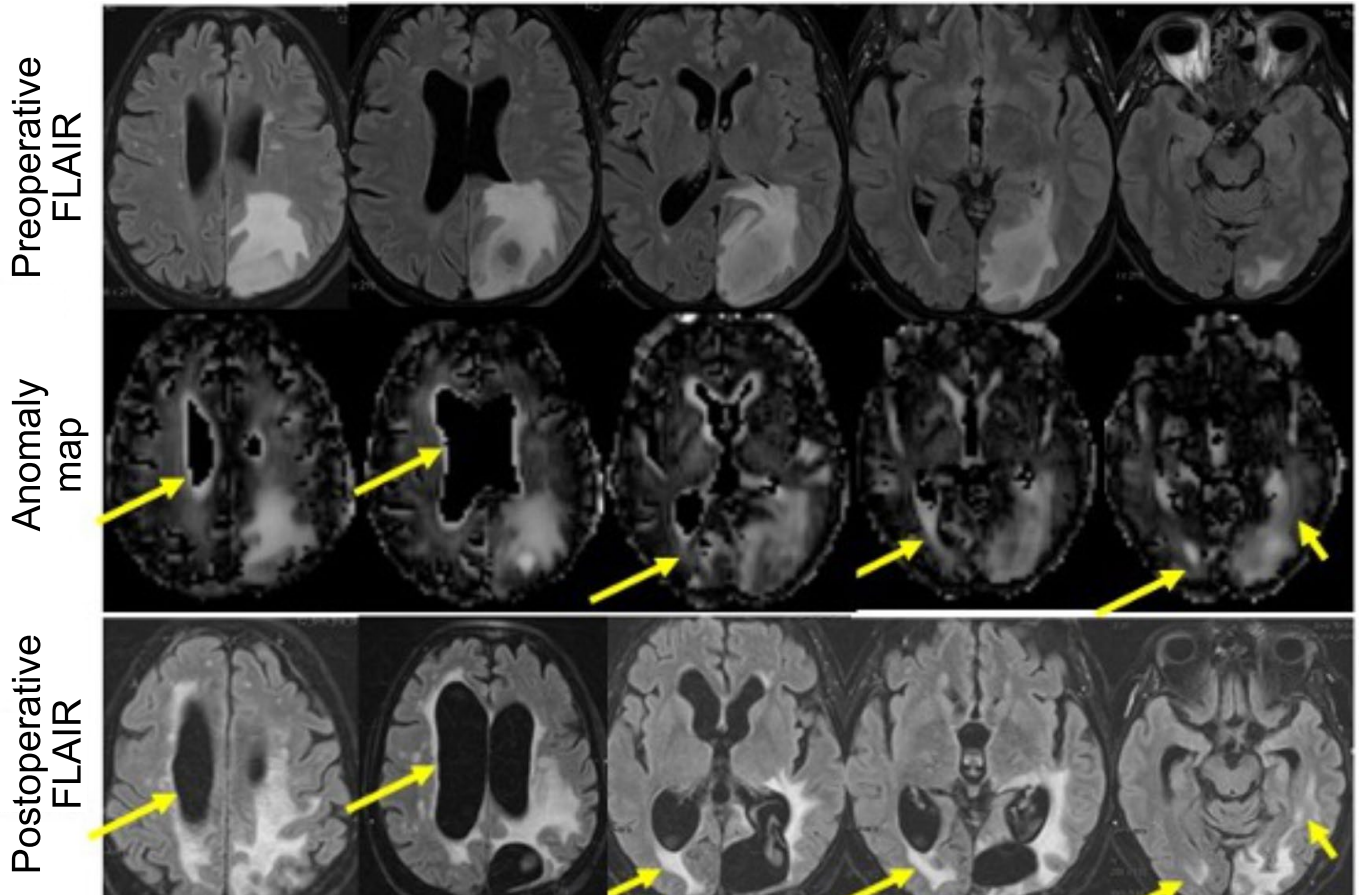
Areas with high anomaly score show up on follow-up scans. Upper two rows: FLAIR-weighted MRI and diffusion anomaly detection-based data obtained preoperatively. Lower row: FLAIR-weighted MRI 18 months later. Yellow arrows mark areas in which tumor recurrence was observed and in which the anomaly score was higher than expected based on the preoperative FLAIR scan.

This visual inspection revealed that the anomaly maps were mostly in concordance with the preoperative FLAIR hyperintense regions, but showed additionally diffusion-anomalous labeled regions in malignant glioma patients: such mismatches were found in 11 out of 14 glioblastoma patients, of which 9 showed partially overlapping areas with FLAIR-weighted MRI follow-up data. Mismatches between diffusion anomaly data and preoperative FLAIR weighted images were observed in 5 out of 10 WHO III glioma patients, of which 3 showed partially overlapping areas with FLAIR-weighted MRI follow-up data. No mismatches were found in WHO II glioma patients. As an exemplary case, preoperative FLAIR-weighted scans and respective anomaly score maps generated by the DAE method are juxtaposed to the 18 months post-operative FLAIR-weighted scans of a glioblastoma patient in Fig. 9. It can be seen that some areas characterize regions with altered diffusive properties not yet visible on preoperative structural scans, but which colocalize to lesions delineated only later on in conventional MRI follow-up data.

## Discussion

Our results demonstrate the potential of dMRI for detecting microstructural anomalies and delineation of brain tumors, offering valuable insights beyond what is achievable with conventional MRI techniques. Here, we will delve into the significance of the study’s findings, highlight the performance of the anomaly detection models, evaluate the distribution of anomaly scores, discuss the binary segmentation results, and provide a qualitative comparison of the models.

### Evaluation of the anomaly map

The evaluation of the DAE anomaly map demonstrated varying performance based on different parameter combinations. The AUC values obtained for different values of the latent space dimensionality and activation functions were presented. Interestingly, models utilizing the sigmoid activation function consistently outperformed those using the linear function. Although the model with the largest latent space dimensionality (m = 12) achieved the highest AUC of 0.820 ± 0.11, even the smallest model (m = 9) performed relatively well with an AUC of 0.804 ± 0.12.

Regarding the RecDiscNet, which does not require residual calculation, the AUC values for different combinations of just-out-of-distribution disturbance appearances were presented. Notably, models trained with $\mu _{Half}$ (representing halving of the mean anomaly distribution) yielded better results, with the “Mix” model achieving the highest AUC of 0.772 ± 0.17. Distribution type and directional dependence seemed to have a minor influence on the results.

### Distribution of anomaly scores

To gain a better understanding of the discriminatory performance, the distribution of anomaly scores generated by the best and worst performing models for $\mu _{Full}$ and $\mu _{Half}$ was analyzed using the density distributions of anomaly scores for different models. Brain matter was mostly scored close to zero by all models, while the scoring of anomalies varied. The best performing model (d = 12) exhibited a strong separation of classes, despite achieving a lower AUC than the best DAE model. Conversely, the worst performing model (d = 1) showed no clear separation between anomalies and healthy brain tissue.

### Binary segmentation

In terms of binary segmentation, the performance of the DAE and RecDiscNet models was evaluated using different thresholding methods. With the simplest approach, pre-defined threshold, the RecDiscNet achieved the highest Dice score of 0.603 ± 0.24, while post-processing significantly improved the results of the DAE, yielding a Dice score of 0.431 ± 0.19.

Data-driven thresholding methods, such as Otsu and the maximum value of the mean pooled output threshold selection, were also evaluated. The Otsu threshold improved the results for the DAE, achieving a Dice score of 0.501 ± 0.20. The mean pooling threshold demonstrated a data-driven improvement for both model, and the post-processed results were particularly superior for the RecDiscNet.

Finally, an optimal threshold, optimized based on the groundtruth data, was determined. While this threshold determination method is not suitable in a real-world application, it is adequate for a unbiased comparison of the DAE and the RecDiscNet. The RecDiscNet outperformed the DAE, achieving a Dice score of 0.657 ± 0.23.

### Qualitative comparison and visual comparison to follow-up scans

To provide a qualitative assessment, we depicted the output of different models for various tumor sizes. These figures demonstrate the ability of the models to detect tumors of different sizes and highlight the differences between unprocessed, binary, and post-processed images. Notably, the post-processed images showed reduced noise and improved segmentation results for both the DAE and RecDiscNet models. Overall, the RecDiscNet consistently exhibited superior qualitative results compared to the DAE when referencing to the ground truth segmentations of structural scans. The optimized thresholding methods further improved the segmentation quality. However, the less clear distinction between normal and anomalous regions and the uncertainty of the anomaly scoring introduced by the DAE models still seemed to contain additional valuable information about microstructural alterations not yet detectable in conventional MRI, which was revealed only by comparison to follow-up structural MRI scans. In particular, mismatches beween structual scans and diffusion anomalous maps at the time of acquisition were only observed in malignant gliomas, which well complies with the more invasive nature of higher tumor grades. As gliomas infiltrate the brain along white matter structures, they are known to impact on white matter integrity by fiber compression and/or displacement and consecutive fiber disintegration. Diffusion MRI is known to detect early microstructural white-matter alterations by capturing altered diffusivity of water molecules as a consequence of altered microstructural boundaries. Such changes in diffusivity have previously been shown to occur in glioma patients even in brain regions which appear in conventional MRI as ‘normal appearing white matter’^34^.

Diffusion-based anomaly detection methods might be more sensitive for the early delineation of occult tumor infiltration, which could aid to identify future itineraries of tumor progression and early detection of tumor recurrence. Combining diffusion-based anomaly detection with other imaging modalities^35^ may allow to further validate and advance the present findings. Potential drawbacks of the method are however that tumor volumes seemed to be rather underestimated in areas near the cortex and in deep nuclei. Also, equivocal signal alterations as well as artifacts, especially in mesiotemporal and basal areas will first require further refinement of the method, as well as validation in longitudinal prospective studies with larger sample sizes is certainly needed, before it might become applicable in the clinical context.

## Conclusion

This study demonstrates the efficacy of deep learning-based anomaly detection methods for the delineation of glioma lesions in diffusion-MRI data. It further highlights the potential of dMRI as a valuable imaging modality for enhancing brain tumor analysis, potentially providing insights into early microstructural changes not yet visible on conventional MRI scans, which could improve prognostication of disease evolution and tumor treatment strategies.

### Supplementary Information

Supplementary Table 1.

## Data Availability

The datasets generated during and/or analysed during the current study are not publicly available due to protection of data privacy of included participants, but not person-related analyzed data are available from the corresponding author on reasonable request.
