# Supplementary material for: Diffusion MRI anomaly detection in glioma patients
Source: Sci Rep. 2023 Nov 21;13:20366. doi: 10.1038/s41598-023-47563-1 (PMC10663596; doi:10.1038/s41598-023-47563-1)
Supplement: Supplementary file 1 — Supplementary Table 1. [file 41598_2023_47563_MOESM1_ESM.pdf]

# Diffusion MRI Anomaly Detection in Glioma Patients: Supplementary Material

## NETWORK ARCHITECTURE

| Block | Layer | Input Channels | Output | Kernel | Stride | Padding |
|---|---|---|---|---|---|---|
| Encoder_1 | Conv3d | 64 | $n \times 64 \times 80 \times 64$ | 3 | 1 | 1 |
| | Conv3d | $n$ | $n \times 64 \times 80 \times 64$ | 3 | 1 | 1 |
| | MaxPool3d | $n$ | $n \times 32 \times 40 \times 32$ | 2 | 2 | 0 |
| Encoder_2 | Conv3d | $n$ | $2n \times 32 \times 40 \times 32$ | 3 | 1 | 1 |
| | Conv3d | $2n$ | $2n \times 32 \times 40 \times 32$ | 3 | 1 | 1 |
| | MaxPool3d | $2n$ | $2n \times 16 \times 20 \times 16$ | 2 | 2 | 0 |
| Encoder_3 | Conv3d | $2n$ | $4n \times 16 \times 20 \times 16$ | 3 | 1 | 1 |
| | Conv3d | $4n$ | $4n \times 16 \times 20 \times 16$ | 3 | 1 | 1 |
| | MaxPool3d | $4n$ | $4n \times 8 \times 10 \times 8$ | 2 | 2 | 0 |
| Bridge | Conv3d | $4n$ | $8n \times 8 \times 10 \times 8$ | 3 | 1 | 1 |
| | Conv3d | $8n$ | $8n \times 8 \times 10 \times 8$ | 3 | 1 | 1 |
| Decoder_3 | ConvTranspose3d | $8n$ | $8n \times 16 \times 20 \times 16$ | 3 | 2 | 1 |
| | Conv3d | $8n + 4n$ | $4n \times 16 \times 20 \times 16$ | 3 | 1 | 1 |
| | Conv3d | $4n$ | $4n \times 16 \times 20 \times 16$ | 3 | 1 | 1 |
| Decoder_2 | ConvTranspose3d | $4n$ | $4n \times 32 \times 40 \times 32$ | 3 | 2 | 1 |
| | Conv3d | $4n + 2n$ | $2n \times 32 \times 40 \times 32$ | 3 | 1 | 1 |
| | Conv3d | $2n$ | $2n \times 32 \times 40 \times 32$ | 3 | 1 | 1 |
| Decoder_1 | ConvTranspose3d | $2n$ | $2n \times 64 \times 80 \times 64$ | 3 | 2 | 1 |
| | Conv3d | $2n + n$ | $n \times 64 \times 80 \times 64$ | 3 | 1 | 1 |
| | Conv3d | $n$ | $n \times 64 \times 80 \times 64$ | 3 | 1 | 1 |
| Output | Conv3d | $n$ | $64 \times 64 \times 80 \times 64$ | 1 | 1 | 0 |

**Table S1.** Architecture of the DAE U-Net. The addition of input channels in the decoder corresponds to an encoder skip connections. $n$ is a parameter and varies the number of filters in the network to control the amount of information for reconstruction. It can be obtained by dividing the mentioned latent space dimension by 8.
